# Size and shape constancy in consumer virtual reality

**DOI:** 10.3758/s13428-019-01336-9

**Published:** 2020-05-12

**Authors:** Rebecca L. Hornsey, Paul B. Hibbard, Peter Scarfe

**Affiliations:** 1 Department of Psychology, University of Essex, Colchester, England; 2 School of Psychology and Clinical Language Sciences, University of Reading, Reading, England

**Keywords:** Virtual reality, Size constancy, Shape constancy, Distance perception

## Abstract

With the increase in popularity of consumer virtual reality headsets, for research and other applications, it is important to understand the accuracy of 3D perception in VR. We investigated the perceptual accuracy of near-field virtual distances using a size and shape constancy task, in two commercially available devices. Participants wore either the HTC Vive or the Oculus Rift and adjusted the size of a virtual stimulus to match the geometric qualities (size and depth) of a physical stimulus they were able to refer to haptically. The judgments participants made allowed for an indirect measure of their perception of the egocentric, virtual distance to the stimuli. The data show under-constancy and are consistent with research from carefully calibrated psychophysical techniques. There was no difference in the degree of constancy found in the two headsets. We conclude that consumer virtual reality headsets provide a sufficiently high degree of accuracy in distance perception, to allow them to be used confidently in future experimental vision science, and other research applications in psychology.

## Introduction

### Distance perception

Accurate visual perception of our surroundings is important for us to interact successfully and efficiently with our environment. To do this, we must be able to estimate the 3D location, shape, and size of objects. Robust measures of perception of these properties in the physical environment have been developed. For example, accuracy in distance perception has been studied using distance bisection (Rieser et al., 1990), verbal estimates (Klein et al., 2009; Mohler et al., 2006), and blind walking tasks (Knapp, 2003; Kuhl et al., 2006). Accuracy in shape and size perception has been examined by allowing observers to compare a seen object to a fixed standard, such as a circular cylinder (Johnston, 1991; Glennerster et al., 1996; Scarfe & Hibbard, 2006), or to a hand-held object (Brenner & van Damme, 1999). These techniques have been implemented in physical space, using either real or computer-generated stimuli, in order to understand the accuracy of human 3D perception using measures of precision and bias. Precision here refers to the variability across multiple estimates for the same stimulus, while bias refers to any systematic deviation from veridical estimates. These methods have also been adopted in virtual space using 3D computer setups, and it has been found that irrespective of the technique used, there does appear to be a misperception of distance, which may present itself as overestimation of near distance, underestimation of far distance, or a combination of both (Foley, 1991; Sinai et al., 1998; Viguier et al., 2001; Li et al., 2011; Higashiyama, 1996; Johnston, 1991; Scarfe & Hibbard, 2006; Yang & Purves, 2003; Patterson et al., 1992; Kline & Witmer, 1996; Chalmers, 1952; Jenkin, 1957).

### Consumer VR for research and other applications

As a method of measuring properties of spatial perception, there are significant benefits of using virtual reality (VR), as it allows precise control of stimulus factors that may be confounded, difficult, or impossible to manipulate in real situations. The ability to separate and test these factors individually allows for a better understanding of the visual system’s processes during the perception of distance. A striking example of this is the expanding virtual room used by Glennerster et al., (2006), where the scale of the virtual environment increases and decreases as the participant navigates through it. These experiments have found that participants are surprisingly unaware of large changes in scale, and allow for the assessment of the role of binocular and motion cues in 3D perception for a moving observer. These changes in scale of the environment, contingent on the participant’s movement, are only possible in VR.

Because of its versatility, VR can be used as a research tool in many fields. In psychology, this includes research into visual perception and spatial cognition (Creem-Regehr et al., 2015; Scarfe and Glennerster, 2015), social interactions (Pan & Hamilton, 2018) and the understanding of conditions such as autism (Kandalaft et al., 2013) and schizophrenia (Freeman, 2008); as well as applications in the fields of education (Freina & Ott, 2015) and therapy (Adamovich et al., 2009). It is now also very affordable, easy to obtain and set up, and is therefore a viable option for many practical applications, for example in training, design, remote working, and the arts. In all applications, within and beyond research, it is important to establish the accuracy of distance perception within VR if it is to be used successfully. Since errors in distance perception have been found across many tasks and setups, including in natural real-world viewing, it is important to know how these errors arise; the extent to which they are exacerbated by properties of VR hardware and software; and the extent to which misperception is specific to particular circumstances. For practical reasons, knowing the extent of any over/underestimation of distance allows for the issue to be mitigated. This is true for both practical applications and for the use of VR in experimental psychology. In studies that address the way in which visual and other cues are used in the perception of 3D space, it is important to understand the nature of any potential biases introduced by the use of VR. It is equally important to know that the virtual environment presented to the participants is perceived as intended by the experimenters. The purpose of the current study was to quantify the accuracy of shape and size settings in consumer VR, which depend on the perception of absolute distance perception.

We measured the accuracy of shape and size constancy in two consumer VR systems: the Oculus Rift and the HTC Vive. The factor which defines these as being *consumer* devices is that the target audience is individuals who would use them for leisure, business, education, or health-care purposes for example; in contrast to being a dedicated research facility. VR systems for research purposes may have a precise and extensive set-up process, often also accompanied by calibration for the individual observer. Ensuring factors such as the inter-ocular distance, viewing position, lens effects, screen resolution, and accommodation distance are all correct can be a time-consuming task. Those using these devices for leisure or non-research applications are unlikely to be able to spend as much time ensuring these features are accurate, and thus consumer devices should be examined for robustness in the absence of these calibrations. Equally, consumer-type VR is increasingly common in all fields of psychological research, and it important to establish that it can be used with confidence without the need for long and laborious calibration procedures.

### Biases in distance perception

**Fig. 1:**
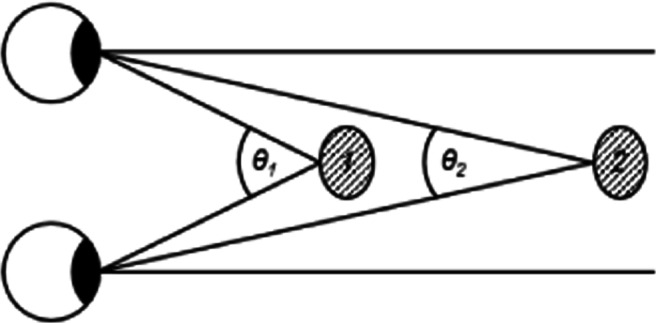
The vergence angles of the two eyes when focusing on two different objects: the angle *θ*_1_ is bigger for object *1* than angle *θ*_2_ when converging on object *2* at a further distance

There are many sources of depth information in the physical world and in VR, including both monocular and binocular cues. Monocular cues are those which depend on the information available from one eye. These include simple features such as size, brightness, texture, and perspective, as well as motion parallax. Motion parallax can be useful in determining the absolute distance to an object (Bishop, 1989). Binocular cues are those which utilize the information from both eyes, such as binocular vergence and retinal disparity. When an observer is fixating on an object, the difference in the direction of the gaze of the eyes, known as *binocular vergence*, can be used to determine absolute distance. When fixating on a close stimulus, the vergence angle is larger than when looking at a stimulus that is further away (Fig. 1). Binocular disparity refers to the differences in the two retinal images for a given vergence state; these differences relate directly to the 3D structure of the environment, and are therefore a valuable source of depth information.

Viguier et al., (2001) investigated the relationship between the cues of vergence and accommodation, and the perception of distance in near space with a series of tasks, including verbal estimates, bisection, and equal- and double-distance settings in real space. They found that observers underestimated the distance of targets beyond 50 cm, but for closer targets distance perception was accurate; the authors noted that this range corresponds to that of arm’s length, a range within which accurate distance perception is needed for effective motor co-ordination (Paillard, 1991).

In the context of VR, it is important to test which factors might contribute to additional inaccuracies in perception, over and above the biases found in the natural environment. Bodenheimer et al., (2007) compared distance judgments via a bisection task in real space, real space with field-of-view-restricting goggles, and in virtual replicas of real space. The idea here was that a restricted field of view may be one of the factors contributing to distance misperception in VR. Two distances (15 and 30 m) were tested, so that the true bisection results should have been 7.5 and 15 m, respectively. Each type of environment (real or virtual space) had an outdoor and indoor setting, tested to increase reliability and external validity of the results. Participants were least accurate in the virtual environments. Accuracy was higher for the closer distance than far: if the midpoint was set to be closer than the actual midpoint, this would indicate that the near space was overestimated relative to far space. The opposite trend was found however; there was an underestimation of the far space section between the mid-point marker and the 15- or 30-m stimuli (referred to as expansion, or *anti-foreshortening*). In addition, estimates in the natural viewing condition of real space were more precise, and the greatest underestimation of the mid-point overall occurred in the virtual environments. These results are slightly different from those found in the previous study in physical space (Purdy and Gibson, 1955), however these discrepancies could be due to methodological differences. The authors concluded that a reduced field of view in head-mounted displays (HMDs) was not the cause of the misperception of distance. This was supported by Knapp and Loomis (2004) who restricted the vertical field-of-view and found this to have no effect on distance estimation, but instead concluded that this error in distance perception may be the result of a number of relatively subtle factors, including the display resolution, dynamic range, and rendering fidelity, combining to produce an overall composite error.

**Fig. 2:**
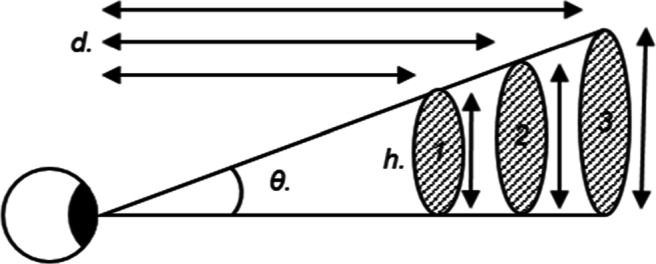
Eye observing three stimuli, where *θ* is the same viewing angle for each; *h.* are the heights of each the object; *d.* are the distances at which each object size would result in the same retinal image. The *d.* to *1.* is shorter than the *d.* to objects *2* and *3*, but because the size of *1.* is smaller than the others also, it results in the same viewing angle for each object at each distance

**Fig. 3:**
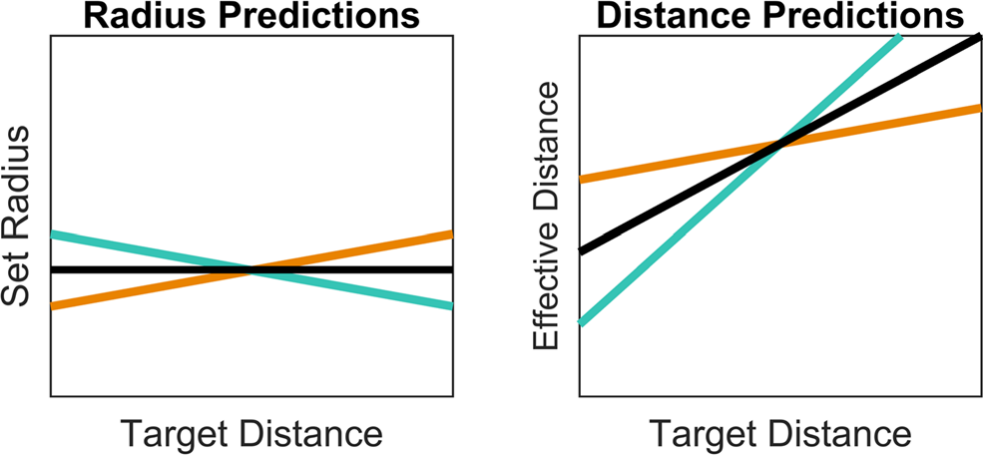
Two sets of possible trends in how setting might vary with distance, and the interpretation of these in terms of misperception of distance. The *Radius Predictions* shows the trend of true constancy in *black*, with an intercept of what the radius of the physical stimuli is, and slope of 0; under-constancy, the prediction based on prior research, is shown in *orange*, in which settings are made too small at close distances and too large at far distances. The opposite possible trend, of over-constancy, is shown in *turquoise*, with setting being too large at close distances and too small at far distances. The *Distance Predictions* shows the possible trends for the effective distances from the radius settings with accurate perception of distance having an intercept of zero and slope of one in black; underestimation shown with a slope of less than 1 in orange and overestimation with a slope of greater than 1 in turquoise. Predictions have been made with a non-zero intercept, since accurate settings tend to be made at an intermediate distance

The results from distance bisection tasks and other experiments (Creem-Regehr et al., 2015) show that in certain areas of space, distances appear to be either under- or over-estimated. If distance is misperceived, then a corresponding misperception of shape and size is also expected. Consequently, measuring the perceived shape and size of stimuli is a method of indirectly estimating perceived distance. Constancy refers to the perceived properties of an object, such as its size and shape, remaining unchanged when there are changes in viewing conditions such as its distance from the observer. If a physical ball is moving through space, or is first shown at a close and then a far distance, with unchanging physical dimensions, then the perception of these dimensions should match. However, there is evidence for a mismatch of the perception of these qualities and the true values in both real space and for binocularly viewed, computer-rendered stimuli. As can be seen in Fig. 2, multiple different combinations of object size and distance will result in the same 2D image being projected onto the retina. If the distance is not known, or is mistaken, then the properties of the object may be misperceived, due to the infinite combinations of size and distance, which result in the same viewing angle. If the object’s dimensions are wrongly identified due to a misperception of its distance, it is possible to work out the distance at which the retinal images perceived by the participant would correspond to the correct physical object dimensions. This approach can be referred to as a *constancy task* and is an indirect method of assessing distance perception. One method of testing this phenomenon involves setting a dimension of a stimulus to match that of another object, which can be compared visually or haptically, across a number of distances. For example, an observer may be asked to set the apparent shape and size of a viewed ellipsoid to match a hand-held tennis ball (Brenner & van Damme, 1999). If true constancy was achieved, then the same radius settings would be made across distances, as shown in Fig. 3(black line in *Radius Predictions*, slope of 0). Translating this into the effective distance observed would cause the perceived and actual distances to be the same (black line in *Distance Predictions*, slope of 1). A different possible outcome would be a progressive over-setting of the radius, such that it increases with presentation distance (shown by the orange line). This would mean that the distance perceived increases at a lesser rate with the distance presented than it should. The opposite possible result is shown by the turquoise lines; progressively smaller settings of the radius with distance would be consistent with perceived distance increasing with the presented distance at a greater rate than it should. Evidence from previous experiments (Johnston, 1991; Glennerster et al., 1996; Brenner & van Damme, 1999; Scarfe & Hibbard, 2006) has typically found under-constancy: perceived distance does increase with presentation distance, but at a lesser rate than expected under veridical perception, as indicated by the orange line in Fig. 3. In this figure, both under- and over-constancy outcomes have been plotted with a non-zero intercept. This is because participants tend to perceive intermediate distances accurately.

Using a physical reference object, which cannot change in its properties (size or shape), has the advantage of providing an external reference, experienced through a different sensory modality, which is therefore not affected by bias in the visual perception of 3D space. This technique has been shown to be a robust measure of distance misperception (Brenner & van Damme, 1999). In a carefully calibrated, 3D screen display, participants were presented with ellipsoidal surfaces. Participants adjusted the size and depth of the ellipsoid until it matched a tennis ball they held in one hand. They then held the tennis ball out in front of them to indicate its perceived distance. As presentation distance increased, so did indicated distance, although indicated distance was gradually more underestimated with increasing presented distance. As the distance of the virtual ellipsoid increased, the settings of size and depth also increased, which the authors concluded was due to participants misestimating the distance to the stimuli. These findings support the predictions shown by the orange lines in Fig. 3. The scaling technique showed an overestimation of perceived space at the near-distance trials, and an underestimation of perceived space at the further distances. This is consistent with the previously discussed compression found to occur in a range of different environments, including the real world (Smallman et al., 2002) and when using HMDs (Ponto et al., 2013; Plumert et al., 2005).

### Shape and size constancy

The geometry relating perceived size and distance to the retinal image is shown in Fig. 2. The retinal size of the image, *θ*, is the same for each object, shown here at three distances. The relationship between *θ* and the distance to the object (*d*) and its height (*h*) is given by:

1 $$ tan(\theta ) = \frac{h}{d} $$

In our experiment, we used the same task as Brenner and van Damme (1999), in which observers were required to match the shape and size of a virtual ellipsoid to those of a hand-held tennis ball. We can distinguish between the *rendered* size and distance of the object (*h*_*rendered*_ and *d*_*rendered*_), as geometrically simulated in VR, and the corresponding *perceived* properties (*h*_*perceived*_ and *d*_*perceived*_). These are related to the retinal image size, and to each other, as follows:

2 $$ tan(\theta ) = \frac{h_{rendered}}{d_{rendered}} =\frac{h_{perceived}}{d_{perceived}} $$

This can then be rearranged to express the perceived distance in terms of the rendered distance and size, and perceived size:

3 $$ d_{perceived} = \frac{d_{rendered} .h_{perceived}}{h_{rendered}} $$

This transformation allows for the perceived distance to be calculated, assuming this to be the source of the error in perceived size. We do this by using the known values of *d*_*rendered*_ and *h*_*rendered*_ and assuming that *h*_*perceived*_ accurately matched the felt size of the hand-held reference ball.

The left and right eyes’ images when viewing an object depend on the observers’ interpupillary distance (IPD) as well as the 3D shape and location of the object. In our experiments, the IPD of individual participants was not measured, and a single value of 63 mm, taken to represent the mean of the population (Dodgson, 2004), was used for all participants. This decision was made to reflect that in most research and other applications, the IPD will not be measured or controlled for, and the purpose of this study was to measure the accuracy of 3D vision under typical conditions. Even in situations in which the viewing geometry is carefully measured and calibrated, the IPD may be fixed at an average value, so that it conflicts with that of most observers (Glennerster et al., 2006).

In both headsets, there is the option to minimally adjust the IPD (stepped and not continuous control), however this was kept constant throughout the trials because casual users are unlikely to adjust these correctly to their own measurements (few if any members of the public will accurately know their own IPD) and some consumer HMDs on the market do not allow for this manipulation. If anything, users might adjust the headset IPD to maximize comfort, but this does not guarantee that the set IPD will match their physical IPD. For example, observers may simply set just enough binocular parallax to create a stereoscopic effect, so called *micro-stereopsis* (Siegel & Nagata, 2000). By keeping the IPD constant, the results obtained would be generalizable to other headsets with similar specifications and to the users who do not know and/or correctly adjust to their own measurements. The effects of a mismatch between the actual and assumed IPD can be calculated geometrically, and depends on the effective distance of the screen from the participant’s eyes (Drascic & Milgram, 1996). In practice, however, the effect on perception is expected to be much reduced in comparison with geometric predictions, since vergence is a rather poor cue to absolute distance (Collewijn et al., 1991). For example, changes in perceived distance with vergence distance show a very shallow slope (Foley, 1980; Johnston, 1991), and a great deal of variability between participants (Gogel, 1977; Morrison & Whiteside, 1984; Richards & Miller, 1969). It has been proposed that, rather than using vergence as an absolute cue to distance, changes in vergence are used as a cue to relative distance, or that the use of vergence as an absolute distance cue is poorly calibrated (Brenner & Van Damme, 1998). As such, biases in distance arising from the mismatch between assumed and actual IPD are likely to be much less severe than predicted geometrically, leading to greater consistency between participants than might otherwise be found. Finally, even with complex photometric calibrations methods used in a lab setting, the calibration is carried out for a single camera separation; for example single IPD (Gilson et al., 2011; Scarfe & Glennerster, 2019). Therefore, using a fixed IPD in the present experiment allowed better generalization to lab-based studies.

Similarly, it is possible, in principle, that biases in the geometrical rendering of the stimuli could have been introduced by the hardware or software used, such that the images presented did not accurately reflect the intended projected images. Again, while this is something that can be measured directly (Gilson et al., 2011), our intention here was to measure the accuracy of perception when VR is used by consumers, or in typical research applications, without this lengthy calibration.

### Current study

The aim of the current study was to measure the accuracy of distance perception in two consumer VR devices using shape and size constancy tasks. Two devices, the Oculus Rift and the HTC Vive, were used in order to assess the generalizability of our results. A difference in results obtained from the two devices was not expected to be found, more so this was a replication in two of the main competing consumer VR devices available on the market. While, on the face of it, the specifications of the headsets are similar, there are in fact some important key differences which could affect the percept of distance, depth, and shape. For example, the lens systems of the two headsets differ. Additionally, anecdotally, one of the authors (PS) has collaborated with industrial partners who rendered large warehouse-size rooms in VR and have reported users experiencing differences in the perception of scale. Thus, while a difference between headsets seemed unlikely, it was a possibility. Comparing two headsets resulted in use collecting twice the amount of data; it allowed us to (1) compare two of the main competing consumer VR devices available on the market (rather than less widely adopted devices) and (2) replicate our findings across the two headsets.

An ellipsoid was shown to participants at randomized distances within near-space; the task was to match the shape and size of the visual ellipsoid to that of a tennis ball, using buttons on a controller to change the depth and size. Based on previous research, it was expected that as presentation distance increased, so too would the size and depth settings, consistent with a progressive underestimation of distance. Furthermore, we expected size and depth measurements to be positively correlated, if variation in these results from the same underlying misestimation of distance. Misestimates of shape and size were used to infer the accuracy of distance perception in the two consumer VR systems. Our overall goal was to assess the degree of shape and size constancy in consumer VR in comparison with the results of previous psychophysical experiments. Thus, while we expect a degree of under-constancy in both shape and size perception, our aim is to assess the extent to which this might be exacerbated by the use of consumer VR, and the implications this might have for research that makes use of VR.

## Methods

### Participants

Opportunity sampling of students from the University of Essex was used to recruit 40 participants, all of whom were naive to the background and hypotheses of the study. The age ranged from 18 to 49 years old, with a mean (standard deviation) of 21.8 (5.6), with 25 males and 15 females. The average completion time was 45 min and all participants had normal or corrected-to-normal vision.

### Apparatus

The experiment was conducted in two VR devices—the HTC Vive and the Oculus Rift—and ran on a PC with an NVIDIA GeForce GTX 1060 graphics card. The IPD was set to the average of 63 mm (Dodgson, 2004). The associated Rift Touch controllers and Vive Motion controllers were used by participants, along with a standard tennis ball with a 3.5-cm radius. Display specifications of the two headsets are summarized in Table 1.

**Table 1:** Display properties of the two VR headsets

Device	HTC Vive	Oculus Rift
Display resolution per eye	1200 x 1800 pixels	960 x 1080 pixels
Field of view (HxV)	110*x*113°	94*x*93°
Pixel size	6.2 arc min	5.2 arc min
Lens	Fresnel	Hybrid Fresnel
Refresh rate	90 Hz	90 Hz

### Stimuli

Stimuli were created and presented using Unreal Engine 4.18 and within the application, the *X* axis represents the horizontal left/right plane, *Z* vertical up/down, and *Y* forward/backward.

Either the left or right controller could be used by participants to modify the stimuli. In the Rift controller, the face buttons, A/B on the right controller and X/Y on the left, changed both the width and height (*X*- and *Z*-axis) and the grip and trigger changed the shape of the ellipsoid by scaling the *Y* dimension of the ellipsoid. On the Vive controller, the four D-pad buttons achieved the same results.

One ellipsoid was visible throughout the experiment. A black and white, random dot image was used as the ellipsoid material to provide a surface texture. In each trial, the ellipsoid was presented at a randomized distance between 40 and 100 cm from the participant along the *Y* -axis, and the size and depth of the ellipsoid were also randomized to values of between 0.5 and 2 times the starting *X*, *Y*, and *Z* values of 7 cm. The minimum and maximum values that could be set for each dimension were 1 cm and 50 cm, respectively.

All stimuli throughout the experiment were presented at eye-height for the individual participant, on a background with no environmental cues that could have influenced their estimates (Lappin et al., 2006): a clouded sky was rendered in full 360° and the ground plane was untextured. The far distance of the sky means that it was rendered with an optical angle disparity of zero, while the untextured ground plane provided no disparity information.

### Task and procedure

**Fig. 4:**
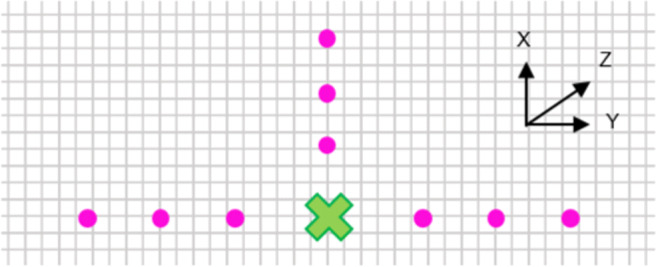
View from above of the correct position for a participant to position themselves marked as a *cross*. The closest of each of the three sets of balls hide the further two in each set

To ensure that the participant was in the correct position in the room, a brief calibration process was completed before the task: two sets of spheres (with the same visual attributes as the target ellipsoid) positioned along the *X*-axis and one along the *Y* -axis were all aligned so that when in the correct position, the closest sphere in each set occluded the two spheres behind. The participants were required to find the correct area within the room for this, which is shown in Fig. 4. All visible spheres could be moved upwards and downwards until they were at eye-height for the participant. These spheres were then removed by a key-command from the experimenter, leaving only the target ellipsoid visible.

The participant grasped the controller in one hand and the tennis ball in the other. The ellipsoid visible in the headset always had the same *Z* coordinate as the participant’s eye-height, which was established during the calibration task. In each trial, the participant altered the appearance of the virtual ball to match that of a tennis ball. A press of any of the buttons on the controller would manipulate the size or depth to increase or decrease at a rate of 1 cm per s.

Once the participant decided that the virtual ellipsoid matched the physical ball, a verbal indication to the experimenter was given so that the size, depth and distance measurements could be recorded. Following this, the next trial began and the distance, initial size, and depth were all randomized. This was repeated until 100 trials were completed.

## Results

**Fig. 5:**
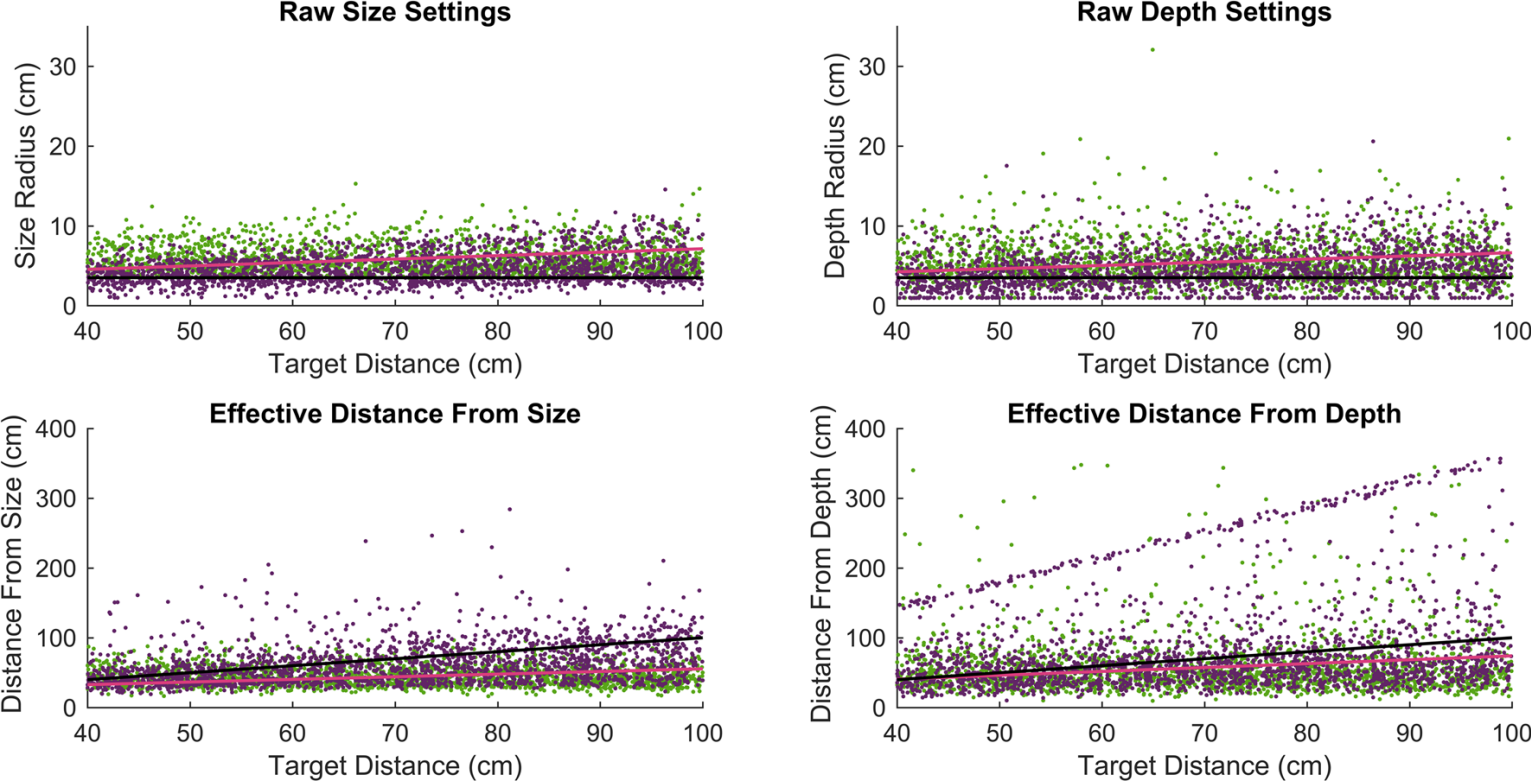
Raw settings for all participants, for the HTV Vive (*green*) and Oculus Rift (*purple*). The *top row* shows the actual radius settings for size and depth. The *bottom row* shows the calculated scaling distance in each case. *Pink lines* superimposed are the predictions from the linear regression model that was fit to the data and *black lines* are accurate performance

The raw data for all participants are plotted in Fig. 5. The data were initially analyzed using one linear mixed-effects model for each dependent variable to assess how size and depth settings were affected by distance. Under perfect scaling, we would expect no effect of distance on these settings. Based on previous studies, however, we expect imperfect scaling, such that both settings will tend to increase with distance, as both size and depth are progressively underestimated. Data from the two headsets were combined and analyzed with two models, with either size or depth as the dependent variable, and object distance as a linear covariate, headset as a categorical factor, and their interaction, included as fixed effects predictors. Random effects across participants were also included. For each dependent variable, we considered two possible random-effects structures (random intercepts only, or random intercept plus the repeated measures factor of distance). The preferred model was chosen as the one having the lowest AIC. AIC values for both models are shown in Table 2 for each dependent variable. In each case, the model with distance as a random factor produced a better fit than the model with only random intercepts, and this structure was therefore used in both cases. However, our conclusions (the direction, size, and significance of our effects) were not affected by this choice of model.

**Table 2:** AIC goodness-of-fit measures for the two models (with or without random distance slopes) for the two dependent variables)

Formula	AIC
size ∼ distance + headset + distance * headset + (1 | participant)	11808
size ∼ distance + headset + distance * headset + (1 + distance | participant)	10591
depth ∼ distance + headset + distance * headset + (1 | participant)	17178
depth ∼ distance + headset + distance * headset + (1 + distance | participant)	16994

In both cases, the model with random slopes gave a better fit, as indicated by the lower AIC values

The models are summarized in Table 3 for size settings and Table 4 for depth settings. In both cases, the intercept was less than the expected value of 3.5 cm, indicating a small overestimation of the radius at near-viewing distances. Settings increased significantly with increasing distance. These results represent a failure of perfect constancy, but are consistent with previous results (e.g., Johnston 1991; Brenner & van Damme 1999; Scarfe & Hibbard 2006). There was no effect of headset, and no headset-by-distance interaction. There was thus no evidence of a difference in distance scaling between the two headsets.

**Table 3:** Results for the size settings

Predictor	Estimate	SE	t	DF	p	Lower	Upper
Intercept	2.821	0.37627	7.4966	3996	8.0224e-14	2.083	3.5584
Headset	− 0.35942	0.53249	− 0.67497	3996	0.49973	− 1.4034	0.68456
Distance	0.04319	0.0072636	5.946	3996	2.9822e-09	0.028949	0.05743
Headset-by-distance	− 0.012486	0.010275	− 1.2151	3996	0.22439	− 0.032631	0.0076596

Size settings increased with increasing distance, indicating incomplete constancy. There was no effect of headset, and no headset-by-distance interaction. These results indicate no difference in size constancy in the two headsets

**Table 4:** Results for the depth settings

Predictor	Estimate	SE	t	DF	p	Lower	Upper
Intercept	2.6421	0.41986	6.2928	3996	3.4529e-10	1.8189	3.4653
Headset	− 0.60032	0.59544	− 1.0082	3996	0.31342	− 1.7677	0.56708
Distance	0.039812	0.0069159	5.7566	3996	9.2286e-09	0.026253	0.053371
Headset-by-distance	− 0.0054208	0.0097963	− 0.55335	3996	0.58005	− 0.024627	0.013785

Depth settings increased with increasing distance, indicating incomplete constancy. There was no effect of headset, and no headset-by-distance interaction. These results indicate no difference in depth constancy in the two headsets

A post hoc note was made of participants who moved or tilted their head to the side (while maintaining the original position of the chair) as these could be classified as using motion parallax. It was found that three participants using the Rift and six using the Vive used this additional cue and after running the same regressions as before, no significant results were found. Using this cue did not aid accuracy in either device. It should be noted that, in this experiment, the role of motion parallax was directly assessed. However, this can be achieved both through tracking head movement data from the headset and experimentally manipulating the degree of motion required of the participants.

For each trial, the effective size and depth distances were calculated, following Eq. 3. A linear scaling of distance was used for each since, although binocular disparity scales with the square of distance, other cues (such as texture) scale linearly. These were analyzed with mixed-effects models, again with distance, headset and their interaction as fixed factors, and distance and intercept as random effects. This reanalysis allows us to calculate the degree of constancy in each case. A zero slope for distance would indicate that participants were not taking distance into account at all in making their settings, while a slope of 1 would indicate perfect scaling. Slopes of 0.38 for size (shown in Table 5 and 0.56 for depth (Table 6 were found, indicating incomplete scaling. The distance scaling calculations were performed on the basis of a linear scaling of depth with distance. For binocular disparity, in contrast to other cues, we know that perceived depth scales with the square of distance. With this in mind, scaling distances were recalculated to take this into account. The result was a slope of 0.71. While this represents better constancy, it is less consistent with the slope estimated for the perception of size. This is likely to reflect that other cues, including texture, were also present in the stimuli, and these scale with distance, rather than its square. The intercept (the effective perceived distance if the rendered distance was zero) was close to 18 cm in both cases. This combination of intercept and slope mean that close distances tend to be overestimated, and far distances underestimated. The crossover distance at which rendered and effective distance were equal was around 30 cm for size settings and 40 cm for depth settings.

**Table 5:** Results for the scaling distances calculated from the size settings

Predictor	Estimate	SE	t	DF	p	Lower	Upper
Intercept	17.756	4.8614	3.6524	3996	0.00026318	8.2245	27.287
Headset	7.8532	6.8807	1.1413	3996	0.2538	− 5.6368	21.343
Distance	0.37739	0.067201	5.6158	3996	2.0898e-08	0.24564	0.50914
Headset-by-distance	0.13453	0.095099	1.4146	3996	0.15726	− 0.051918	0.32098

Effective distance increased with rendered distance at a rate of 38 %, indicating incomplete scaling. There was no effect of headset, and no headset-by-distance interaction. These results indicate no difference in size constancy in the two headsets

**Table 6:** Results for the scaling distances calculated from the depth settings

Predictor	Estimate	SE	t	DF	p	Lower	Upper
Intercept	18.474	9.9706	1.8529	3996	0.063974	− 1.0737	38.022
Headset	9.1321	14.139	0.64588	3996	0.5184	− 18.588	36.853
Distance	0.55612	0.15886	3.5006	3996	0.00046926	0.24466	0.86758
Headset-by-distance	0.18816	0.22504	0.83609	3996	0.40315	− 0.25305	0.62936

Effective distance increased with rendered distance at a rate of 56 %, indicating incomplete scaling. There was no effect of headset, and no headset-by-distance interaction. These results indicate no difference in depth constancy in the two headsets

### Correlation between size and depth

A set of correlations was undertaken on the raw depth and size data from both headsets combined, and a correlation coefficient calculated for each participant. A one-sample *t* test against zero was performed on all correlations for size, and another for depth correlations. The analysis on the size correlations was t(39) = 10.42, *p*< 0.001; for depth t(39) = 9.10, *p* < 0.001. These results show that the variability in the two settings was positively related, as expected if errors in both represent a common misperception of distance.

## Discussion

The purpose of the current study was to use shape and size constancy tasks to infer the accuracy of distance perception in virtual environments for two consumer-ready, uncalibrated HMDs. Participants wore either the Oculus Rift or HTC Vive and observed an ellipsoid within near-space. Their task was to match the size and depth of the ellipsoid to a physical tennis ball they were able to grasp. Accurate results would show constant settings made across the range of distances presented.

**Fig. 6:**
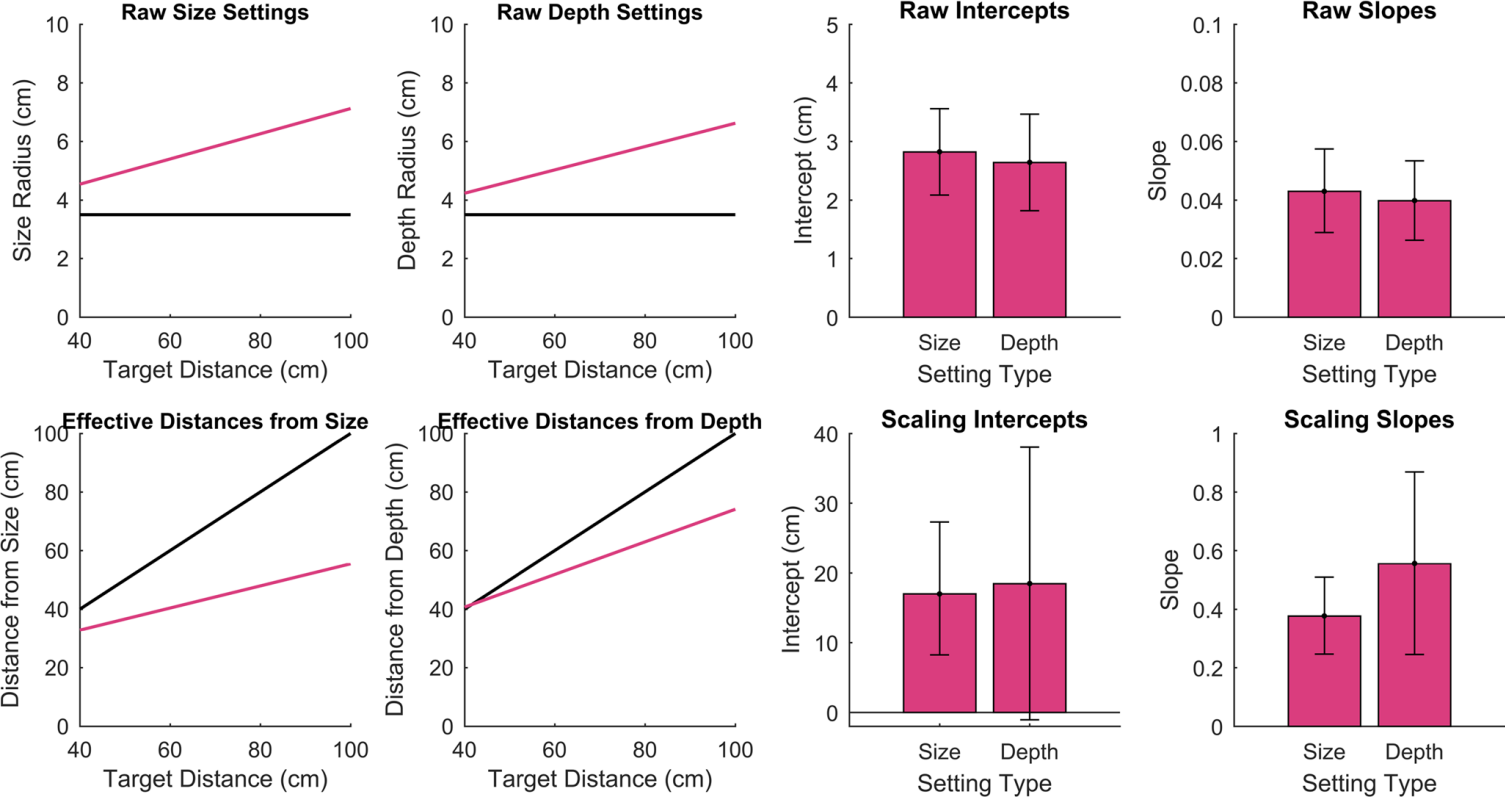
Results of all participants with the *pink line* showing the fitted regression lines. The *black lines* show true constancy, having an intercept of 3.5 and slope of zero in the raw radius settings and an intercept of zero and slope of one in the effective scaled distances. The *bar graphs* show the mean 95% confidence limits for each fitted parameter

Perfect constancy across distances for size or depth was not found in either device. In Fig. 6, it can be seen that as the presentation distance increased, both the size and depth settings also increased and these trends did not differ between the devices. Consequently, scaled distances for both size and depth show a general misperception of distance in all cases. Our results show an overestimation of near distance and underestimation of far distance. Overall, the results show a general under-constancy, with changes in effective distance appearing to be 47 % smaller than expected from the variation in rendered distance.

The radius settings of the original data set (depth and size) should be positively correlated in that as participants set the ellipsoid to be increasing in size (as distance increased, which was expected due to presumed lack of constancy) they would also increase the depth setting (if the same error affected both settings). Correlations showed that generally this was the case, and a *t* test on these showed that the perception of size and depth are related, where the systematic errors made for one are likely to be the cause for the errors in the other.

It is important to note that the degree of shape and size constancy, of around 47 %, is very similar to that found for carefully calibrated 3D displays (Brenner & van Damme, 1999) when a similar task was used. We therefore found no evidence for additional sources of misperception associated specifically with consumer VR. In other studies, distance compression has been found in VR when compared with the real world (Creem-Regehr et al., 2015). These differences may include cognitive effects such as expectations of the room size, rather than any biases introduced by rendering of binocular or motion cues, image quality or field of view (Creem-Regehr et al., 2015). In our case, with very limited visual cues available, compression of the perceived distance range is consistent with the uncertainty of the perceptual information provided (Mon-Williams et al., 2000).

Our study also provides a starting point for future experiments to examine the role of specific factors in accurate perception in VR, such as the development of high-quality graphics, to improve the 3D experience, and how much these additional features might affect distance perception in immersive HMDs. It has been suggested that the visual system may not always need to recover full metric shape information in everyday interactions (Bradshaw et al., 2000). Investigations into which specific cues, such as perspective, texture, binocular disparity, and motion parallax are necessary for producing accurate or aesthetically pleasing displays within VR are therefore important areas for research using consumer VR. There is evidence that perspective and binocular disparities make different contributions to depth and shape perception (Van Ee et al., 2002; Welchman et al., 2005) and that the cues are differentially used at different viewing distances (Hillis et al., 2004; Surdick et al., 1997; Keefe et al., 2011) due to the way in which their relative reliability changes with distance (Watt et al., 2007). The results and methodological setup here can therefore be used to provide a basis to explore these specific cues and their contribution to distance perception in VR.

Overall, our results are consistent with the findings of other studies into constancy within 3D set-ups. There were no specific problems identified for distance scaling in consumer VR, with the expected result being found that observers reported smaller objects at further distances than near. Importantly, we found no systematic differences between the two headsets used, and no evidence for errors in constancy over and above what would be expected when sparse cues to distance are available. We conclude that consumer VR headsets provide a sufficiently high degree of accuracy in distance perception which, despite their low resolution and optical distortion, is comparable to more specialist 3D setups, to allow them to be used more confidently in future experimental vision science, and other applications in psychology.
